# Mild cryoinjury in zebrafish fin induces regenerative response without blastema formation

**DOI:** 10.1111/dgd.12962

**Published:** 2025-02-01

**Authors:** Takafumi Yoshida, Atsushi Kawakami

**Affiliations:** ^1^ School of Life Science and Technology Institute of Science Tokyo Yokohama Japan

**Keywords:** cryoinjury, fin, regeneration, regeneration‐response enhancer, zebrafish

## Abstract

Previous studies have shown that tissue regeneration induces expression of genes that play important roles in regeneration. Recently, several studies have identified regeneration‐response enhancers (RREs) that activate gene expression by tissue injury. Particularly, we showed that RREs contain two transcription factor‐binding motifs: a bHLH transcription factor‐binding motif, an E‐box, and an AP‐1/bZIP transcription factor‐binding motif, a 12‐O‐Tetradecanoylphorbol 13‐acetate response element (TRE). However, the triggers and subsequent signals generated by injury are still unclear. In this study, we analyzed RRE activation using various injury models. Although inter‐ray incisions and skin exfoliation injuries did not activate RREs or regeneration genes, the fin puncture injury activated RREs and several regeneration‐response genes. After fin puncture injury, *msxc* was activated only on the proximal side of the hole where blastema‐like tissue was formed, whereas RREs, *junbb*, and *fibronectin 1b* (*fn1b*) were activated on both the proximal and distal sides, implying that activation of RREs, *junbb*, and *fn1b* is independent of blastema formation. Here, we also established a mild cryoinjury method. After this injury, transient vascular destruction, an increase in cell death, and an accumulation of myeloid cells were observed; however, no major morphological damage was observed. Importantly, *msxc* was not induced by cryoinjury, whereas *fn1b*, *junbb*, and *1.8 k* RRE (−*1.8 kb promoter* of *fn1b*) were activated, suggesting that cryoinjury induces the responses of *fn1b*, *junbb*, and *1.8 k* RRE without forming the blastema. Thus, our study shows that the cryoinjury model and the RRE transgenic (Tg) zebrafish may provide a useful platform for exploring injury signals.

## Graphical abstract

Regeneration‐response enhancers have been recently revealed, but triggers or signals that activate the enhancer are unclear. We performed a comprehensive analysis of the RRE response in various injury models in zebrafish including a newly developed cryoinjury method. Cryoinjury is sufficient for inducing a response of RRE and several regeneration‐induced genes without blastema formation.

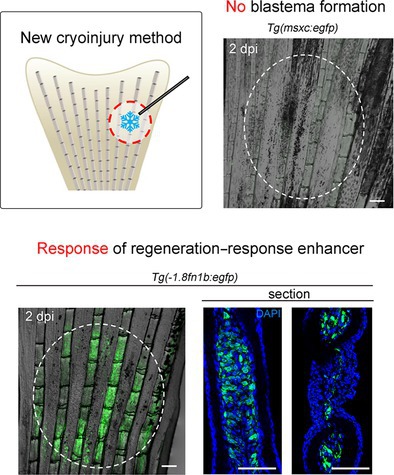

## 1 INTRODUCTION

During evolution, multicellular organisms acquired the ability to maintain tissues and organs, termed tissue homeostasis. A drastic case of tissue homeostasis is seen in tissue regeneration (Cano‐Martínez et al., 2024; Hasegawa et al., 2015; Ishida et al., 2010), but its regulation is poorly understood. Fins of teleost fish and urodele limbs have been used as good models for exploring the mechanism of regeneration (Kawakami, 2010; Kumar et al., 2007; Poss, 2010; Tanaka, 2016).

Research in this field over the past two decades has shown that the expression of a group of genes that may play important roles is induced during regeneration (Poss et al., 2003; Wehner & Weidinger, 2015; Yoshinari et al., 2009). Similar to many other genes, it has been predicted that these genes are regulated by the enhancer elements that exist around the genes. Indeed, a series of regeneration‐response enhancers (RREs) have been identified in recent years (Kang et al., 2016; Pfefferli & Jaźwińska, 2017; Wang et al., 2020). Kang et al. (2016) identified an RRE that works during regeneration of the caudal fin and heart from the zebrafish *leptin b* (*lepb*) locus and showed that this RRE is also activated by finger amputation in mice. Wang et al. (2020) performed a comparative analysis of epigenetic profiling and single‐cell RNA sequencing using two related teleost species, African killifish and zebrafish, and identified another RRE, K‐IEN, near the *inhibin beta a* (*inhba*) gene. Furthermore, we have identified RREs in the promoter region of the zebrafish *fn1b* gene (Tamaki et al., 2023). A comparison of multiple RREs has shown that they commonly contain two transcription factor‐binding motifs, the E‐box, a bHLH transcription factor‐binding motif, and the TRE, the AP‐1/bZIP transcription factor‐binding motif, and that their cooperative action is essential for RRE function. Given that the entity of RREs has become clear, a question is what sort of triggers and subsequent signals activate them.

In addition to tissue amputation, various injury models have been tested to analyze tissue regenerative responses. Cao et al. (2021) used a fin puncture injury, in which a hole was made within the fin. They showed that the blastema markers such as *and1* and *msxc* were expressed on the proximal side of the hole, and that regeneration progressed from the proximal to the distal direction. Further, Chen et al. (2016) developed a skin exfoliation injury model, in which keratinocytes on the skin surface were removed using a cotton swab. Furthermore, a model that provides an incision in the inter‐ray tissue has been reported (Wang et al., 2020); however, it cannot activate the K‐IEN RRE response. In addition, Chassot et al. (2016) reported a severe cryoinjury model. In this method, a cryotome blade cooled with liquid nitrogen was contacted across the fin along the dorsoventral axis. This method caused severe tissue necrosis around the injury site and loss of the entire tissue caudal to the injury, after which the normal fin regeneration process was initiated.

The various injury models that have been tested so far have resulted in either major tissue loss that accompanies blastema formation or no regenerative response. In this study, we established a mild cryoinjury model that does not induce severe tissue damage and consequent loss of distal fin tissue, which was observed in the severe cryoinjury method (Chassot et al., 2016), and performed a comprehensive analysis of various injury models for their ability to activate RREs and regeneration‐induced genes. We showed that mild cryoinjury can induce an RRE response and the expression of several regeneration genes without blastema formation.

## 2 MATERIALS AND METHODS

### 2.1 Zebrafish husbandry and lines used

Zebrafish husbandry and maintenance were performed according to standard procedures. All the animals were handled in accordance with the Animal Research Guidelines of the Institute of Science Tokyo, Japan. All surgical procedures were performed under anesthesia using 150 mg/L tricaine (3‐aminobenzoic acid ethyl ester) (Tokyo Chemical Industry).

### 2.2 Transgenic zebrafish

The following transgenic (Tg) zebrafish were used in this study. *Tg*(*−1.8fn1b:egfp*)^*tyt218*^, hereafter referred as *1.8 k* Tg, carries a 1.8 kb‐upstream sequence of the *fn1b* gene and expresses *egfp* in the blastema in response to fin amputation (Tamaki et al., 2023). *Tg*(*E2S‐miniP:egfp*)^*tyt222*^, hereafter referred as *E2S* Tg, carries a short RRE termed *E2S* and a synthetic *minimum promoter* and expresses *egfp* in the wound epidermis in response to fin amputation (Tamaki et al., 2023). Further, we used *BAC Tg*(*fn1b:egfp*)^*tyt212*^ (Shibata et al., 2018), and *BAC Tg*(*msxc:egfp*)^*tyt240*^ (in this study) for evaluating the regenerative response of epidermis and mesenchymal cells, respectively. For visualization of endothelial cells, red blood cells, and neutrophils, *Tg*(*fli1:egfp*)^*y1*^ (Fukui et al., 2009), *Tg*(*gata1:mrfp*)^*ko06*^ (Fukui et al., 2009), and *Tg*(*lysC:egfp*)^*ko02*^ (Kitaguchi et al., 2009) were used, respectively.

*BAC Tg*(*msxc:egfp*) was generated by the BAC recombineering protocol according to a previously described procedure (Ando et al., 2017; Shibata et al., 2018; Suster et al., 2011). Briefly, the *egfp* gene cassette that also carries the polyadenylation sequence and kanamycin resistance gene was amplified by polymerase chain reaction using the primers listed below (lowercase letters indicate regions homologous to the *msxc* gene) and inserted at the translational initiation site of *msxc* of the BAC clone, CH211‐217G15, by homologous recombination. *msxc egfp* fw, 5′‐ctgctggaccagagtacagttcaggacgactcacatattcttgttgtATGGTGAGCAAGGGCGAGGAGCT‐3′, *msxc egfp* rv, 5′‐ctttgtccgcgcttttgcgctcctgtagttttccttcttgggagagtggcTCAGAAGAACTCGTCAAGAA‐3′.

The iTol2 cassette that carries the *ampicillin resistance* gene and *tol2* arms (Suster et al., 2011) was also introduced into the vector sequence of the BAC clone. The engineered BAC DNA was injected into one‐cell‐stage embryos at 125 ng/mL with 25 ng/mL transposase mRNA. The F1 offspring were identified by fluorescence during the embryonic stage and were confirmed by the EGFP expression in the blastema in the adult stage.

### 2.3 Injury methods

For inter‐ray incision, a 2‐ to 3‐mm incision was made between the fin rays using a thin needle according to the method described by Kizil et al. (2009). For exfoliation injury of the skin, the epidermal surface of the fin was gently rubbed five times with a cotton swab according to the method described by Chen et al. (2016). In the fin puncture injury, a rectangular hole with 300‐ to 500‐μm sides was made using a scalpel according to the method described by Cao et al. (2021). Mild cryoinjury was induced by using a standard stainless‐steel needle (G40‐1202, AS ONE). The tip of the needle was immersed in liquid nitrogen for 90 s and immediately contacted with the fin surface for 15 s. The cooled area was confirmed by the frost on the surface of the fin.

### 2.4 Immunostaining and histological analysis

Zebrafish fins were fixed with 4% paraformaldehyde (PFA) in phosphate‐buffered saline (PBS) at 4°C overnight, subsequently dehydrated with methanol, and stored at −20°C. The samples were rehydrated with PBT (PBS plus 0.1% Tween‐20), equilibrated with 20% (w/v) sucrose, and embedded in OCT compound (Tissue‐Tek, Sakura Finetek). Cryosections were prepared at thicknesses of 16–20 μm.

For immunofluorescence staining, cryosections were gently washed twice with PBS and several times with PBT to remove the OCT compounds. Antibody staining was performed as described previously (Shibata et al., 2016). A 1:1000 dilution of the anti‐GFP antibody (Nacalai Tesque, 04404–26; #598, MBL) was used. The zns5 antibody was used at a 1:100 dilution of the hybridoma supernatant (Zebrafish International Resource Center, RRID:AB_10013796). The sections were counterstained with 4′,6‐diamidino‐2‐phenylindole (DAPI; 0.1 μg/mL, Invitrogen) and mounted with 80% glycerol containing 25 mg/mL triethylenediamine (DABCO, Nacalai Tesque). Images were captured using a confocal microscope (LSM780; Zeiss) or a fluorescence microscope (APX100; Olympus).

### 2.5 Whole‐mount in situ hybridization

Whole‐mount in situ hybridization (ISH) was performed according to the standard protocols (Thisse & Thisse, 2008). The *junbb* RNA probe was synthesized from amplified DNA as a template. Capital letters indicate the T7 promoter sequence.

*junbb* probe F 5′‐atgagtacaaaaatggagcagccgttttac‐3′.

*junbb* probe R 5′‐TAATACGACTCACTATAGGGaactctccgttggttccttcccaggtgatg‐3′.

### 2.6 Detection of apoptosis and quantification

Fins were fixed in 4% PFA in PBS at 4°C overnight, dehydrated with methanol, and stored at −20°C. Apoptosis was detected using the In Situ Apoptosis Detection Kit (Sigma). Briefly, samples were rehydrated with PBTx (PBT plus 0.1% Triton X‐100), treated with 10 μg/mL Proteinase K in PBTx, washed with PBTx, and refixed with 4% PFA in PBS. The samples were further incubated in a freshly prepared 0.1% sodium citrate buffer containing 0.1% Triton X‐100, washed with PBTx, and allowed to react with the terminal transferase dUTP nick end labeling (TUNEL) reaction mixture at 37°C for 2 h. The reaction was terminated by washing the tissues with PBTx. The samples were mounted in 80% glycerol. Images were acquired on a fluorescence microscope (M205FA, Leica). TUNEL cells were quantified as the number of stained cells in 1 × 1‐mm square area using the acquired images. Statistical analyses were performed using Microsoft Excel 2019.

### 2.7 Movies

Fishes were anesthetized with 0.002% tricaine and placed on a 2% agarose gel. Videos of *Tg*(*gata1:mrfp*) at 1 and 3 days after injury (dpi) were captured for 5 s using a fluorescence microscope (M205FA, Leica).

### 2.8 Quantification and statistical analysis

No statistical method was used to determine the sample size. Sample sizes were chosen based on previous publications and experiment types, and are shown in the figure legends. No animal or sample was excluded from the analysis unless the animal died during the procedure. Most assessments were repeated by performing at least three independent experiments. Sample sizes are indicated in the figures or legends.

## 3 RESULTS AND DISCUSSION

### 3.1 Establishment of *Tg*(*msxc:egfp*) that marks the blastema

**FIGURE 1:**
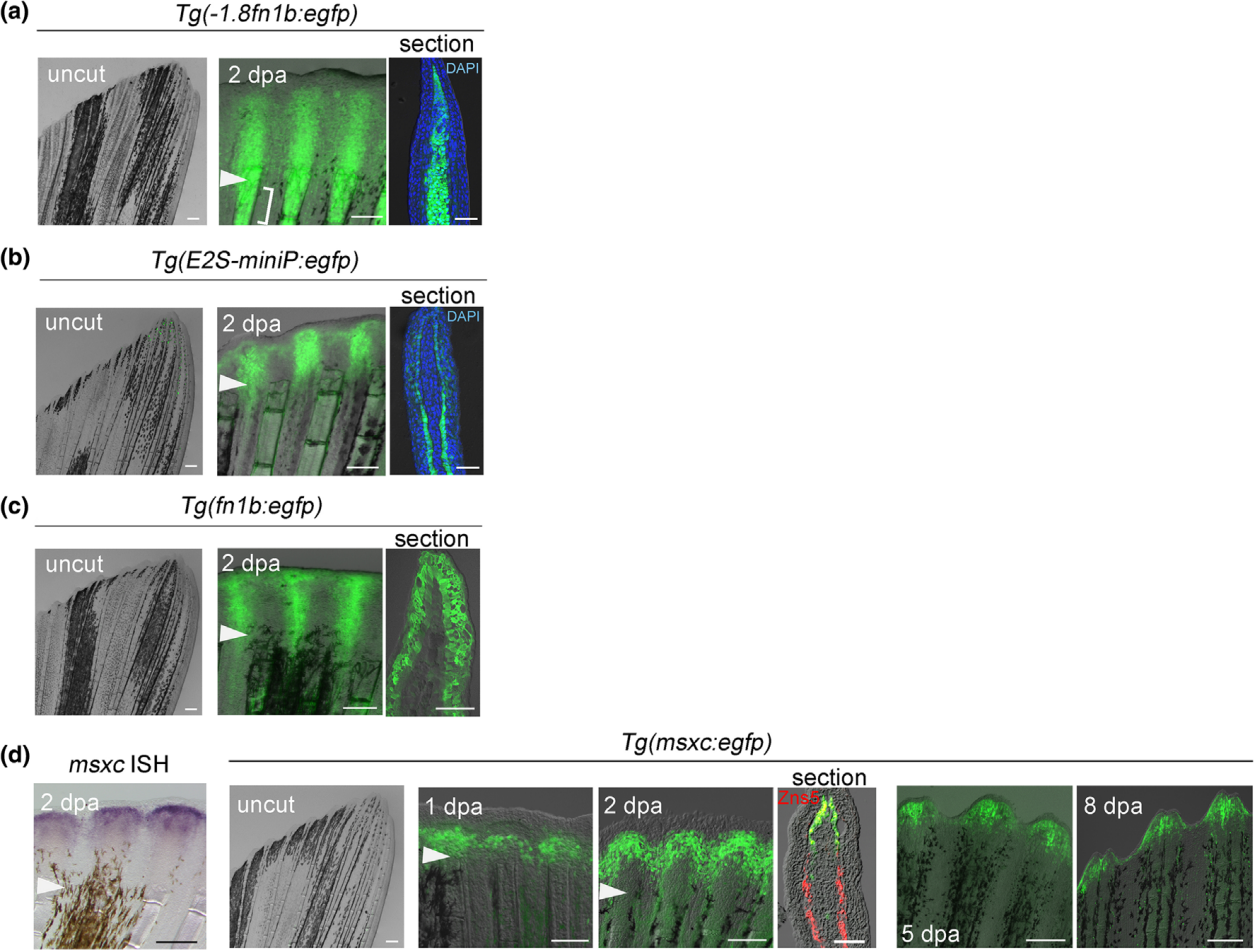
Transgenic (Tg) line used in this study. (a) Response of *Tg*(*−1.8fn1b:egfp*) (*1.8 k* Tg) to fin amputation. The response was observed in mesenchymal cells of regenerating fin. Note that the response extends proximal to the amputation plane (Bracket region). (b) Response of *Tg*(*E2S‐miniP:egfp*) (*E2S* Tg) to fin amputation. The response was observed in the wound epidermis, strongly in the basal layer of the epidermis. (c) Response of *Tg*(*fn1b:egfp*) to fin amputation. EGFP is expressed in the entire wound epidermis including the superficial keratinocytes. (d) *msxc* expression during fin regeneration by whole‐mount in situ hybridization (ISH) (left panel) and by *Tg*(*msxc:egfp*). EGFP expression recapitulates the *msxc* expression, and is seen in the distal region of the blastema. EGFP fluorescence was observed from 1 day post amputation (dpa) to 8 dpa. Note that the *msxc* ^+^ cells do not overlap with the zns5^+^ osteoblasts (section). Arrowheads denote the respective amputation planes. Scale bars, 100 μm (whole‐mount) and 50 μm (sections). *n* > 10, respectively.

We previously generated a number of Tg lines carrying RREs that express EGFP in the epidermis or mesenchymal cells in response to fin amputation (Tamaki et al., 2023). In this study, we focused on the *1.8 k* Tg, which expressed EGFP in the blastema mesenchymal cells, and the *E2S* Tg, which expressed EGFP in the wound epidermis (Figure 1a,b). In addition, we used *BAC Tg*(*fn1b:egfp*) (Figure 1c; Shibata et al., 2018), which induces EGFP expression in the wound epidermis. Additionally, we created a new *BAC Tg*(*msxc:egfp*) that marks the blastema (Akimenko et al., 1995) and enables live imaging of the blastema. In the established Tg line, EGFP expression was observed in the distal region of the blastema (Figure 1d), which agrees with the endogenous *msxc* expression (Akimenko et al., 1995). EGFP^+^ cells that did not overlap with osteoblasts were observed from 1 to 8 days after amputation (Figure 1d).

### 3.2 RREs, *junbb*, and *fn1b* are activated by an injury signal, independent of blastema formation

**FIGURE 2:**
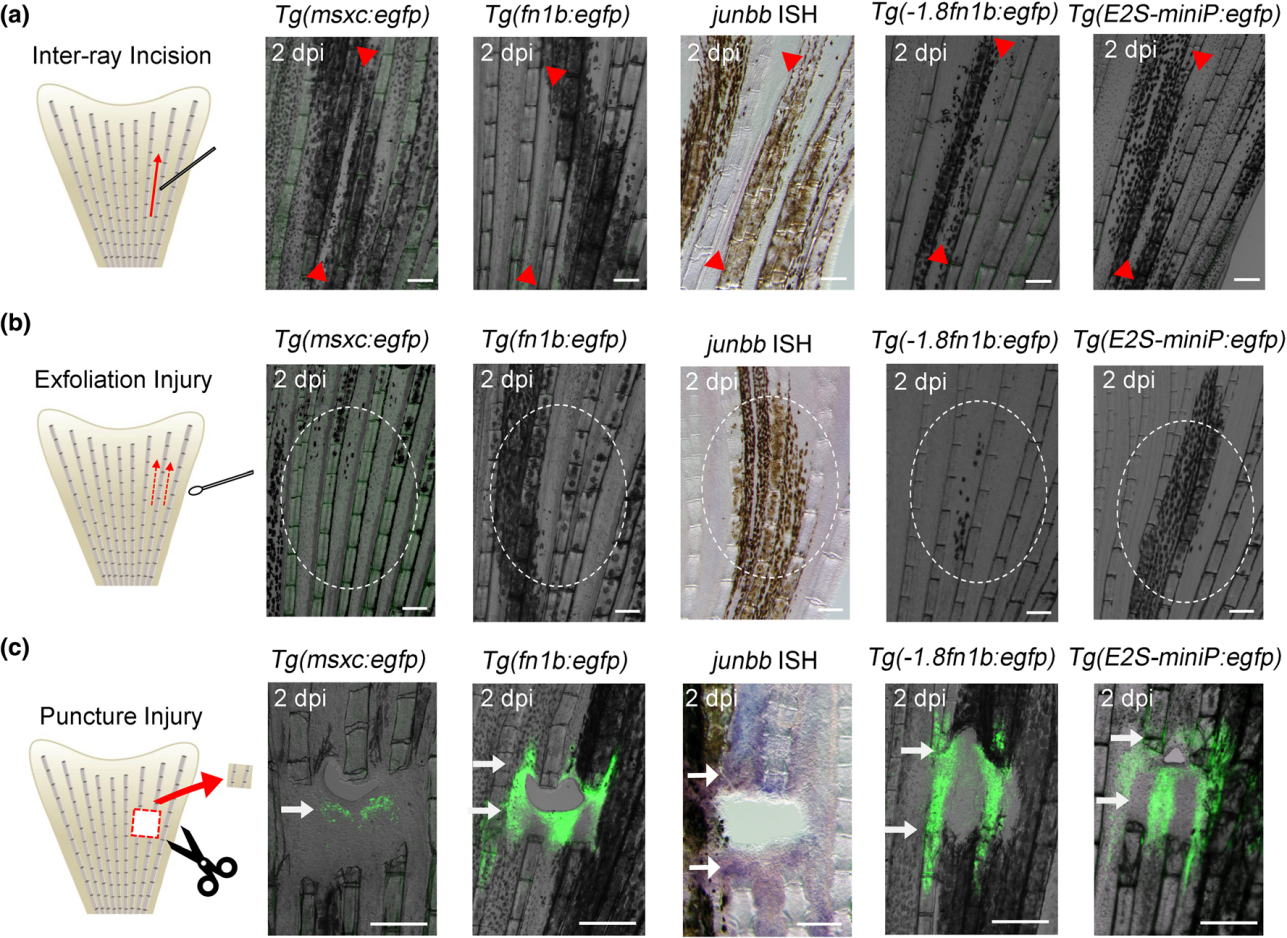
The responses of regeneration‐induced genes and regeneration‐response enhancers (RREs) in various injury models. (a) Responses of RREs and respective genes in the inter‐ray incision. EGFP expression was neither observed in all transgenic (Tg) lines at 2 days post injury (dpi) and at any stage of the healing process. Arrowheads indicate the ends of the incision. (b) Responses of RREs and respective genes in the skin exfoliation injury. EGFP expression was not observed in all Tg lines even with this injury method. Circles mark the injured regions. (c) Responses of RRE and respective genes in the fin puncture injury. Arrows indicate the regions of gene expression or RRE response. The EGFP fluorescence of *Tg*(*msxc:egfp*) was detected in the proximal side of the hole, but not in the distal side. However, *fn1b*, *junbb*, and both RREs showed responses on both proximal and distal sides. *Junbb* expression was detected by in situ hybridization (ISH). Scale bars, 100 μm. *n* = 5, respectively.

Wang et al. (2020) reported that the K‐IEN, an RRE that was identified by their study, was not activated by inter‐ray incision injuries. We tested the responses of the *1.8 k* and *E2S* RREs using this injury model. Similar to that observed in K‐IEN, neither of *1.8 k* nor *E2S* RRE responded to inter‐ray incision (Figure 2a). In addition, the regeneration‐induced genes *msxc* and *junbb* in the blastema (Ishida et al., 2010; Yoshinari et al., 2009) and *fn1b* in the wound epidermis (Shibata et al., 2018) were also not activated by inter‐ray incision. We also tested the responses to skin exfoliation injuries (Chen et al., 2016), but the response of RREs and regeneration genes was not observed (Figure 2b).

Next, we examined the responses by fin puncture, an injury model that makes a hole spanning two or three fin rays in the fin (Cao et al., 2021). In this model, it has been reported that a blastema‐like tissue that expresses *msxc* was formed on the proximal side of the hole, and that regeneration proceeded from proximal to distal direction. As described by Cao et al. (2021), we observed that the *msxc* was expressed only on the proximal side of the hole using the *Tg*(*msxc:egfp*) (Figure 2c). However, in contrast to *msxc*, *junbb* expression was observed on both the proximal and distal sides of the hole (Figure 2c). Similarly, *fn1b* expression was also observed in the epidermis on both sides of the hole (Figure 2c). These results suggest that a signal induced by injury can induce *junbb* and *fn1b* expression in the mesenchyme and epidermis, respectively, but the *msxc* induction in the blastema may involve a different or an additional mechanism. We speculated that *junbb* and *fn1b* expression may represent a primed cellular state induced by an injury signal. Cao et al. (2021) showed that FK506, an inhibitor of calcineurin signaling, induces *msxc* expression on the distal side of the hole. Calcineurin signaling could play a role in forming the *msxc* ^+^ proliferating blastema cells.

We next examined the response of *1.8 k* and *E2S* RREs to the puncture injury and found that EGFP expression was observed in both *1.8 k* and *E2S* RREs, indicating that the fin puncture injury induced the response of these RREs (Figure 2c). Importantly, EGFP expression was induced on both the proximal and distal sides of the hole, suggesting that, like *junbb* and *fn1b*, *1.8 k* and *E2S* RREs are activated by an injury signal, independent of blastema formation.

### 3.3 Development of mild cryoinjury model and evaluation of traumatic effects

In the injury models tested thus far, only injuries that span the fin rays accompanying blastema formation can elicit an RRE response. To verify whether fin‐ray amputation is essential for the RRE response, we sought to develop a new injury model that does not involve fin‐ray amputation. Cryoinjury has been previously used as an alternative method to induce heart muscle regeneration in zebrafish (Chablais et al., 2011). A cryoinjury to the fin was previously attempted by Chassot et al. (2016); however, because they gave a severe cryoinjury that spanned over their entire dorsoventral length, the tissue distal to the injured site became necrotic and was lost, and then normal fin regeneration took place.

**FIGURE 3:**
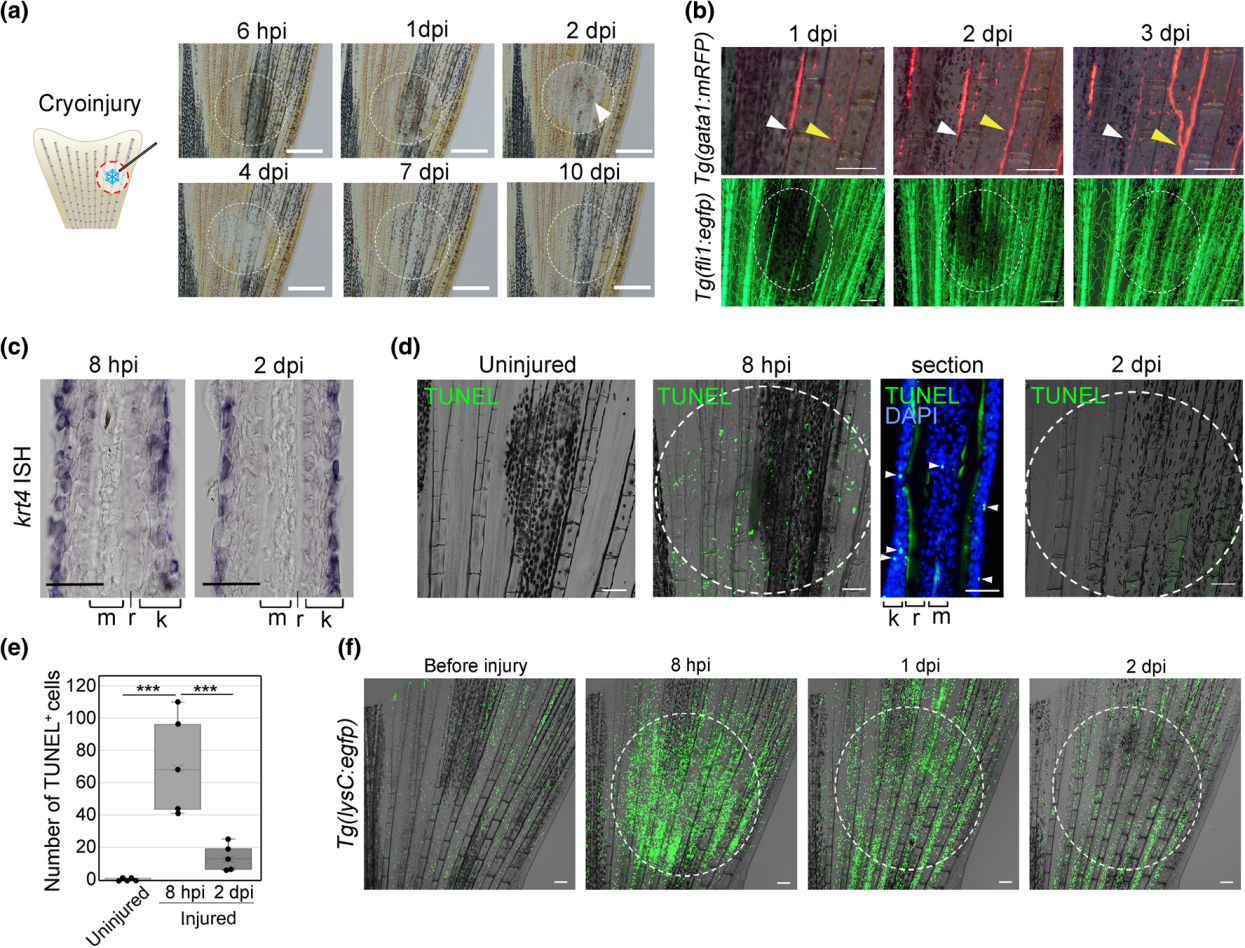
Morphological and histological assessment of traumatic effects caused by mild‐cryoinjury. (a) A schematic illustration of the cryoinjury method and effect on melanocytes. Loss of melanocytes becomes evident by 2 days post‐injury (dpi) and persists up to 10 dpi. Within the injured area, a transient hyperemia is often observed (arrowhead). *n* > 10. (b) Damage to the vascular network after cryoinjury. The blood flow is visualized by *Tg*(*gata1:mrfp*) (upper panels), and the vascular endothelial cells by *Tg*(*fli1:egfp*) (lower panels). Blood congestion was noticeable at 1 dpi, but had almost recovered by 3 dpi (white arrowheads). Reduced blood flow was also observed in the injured area, but recovered by 3 dpi (yellow arrowheads). Consistent with the effect on blood circulation, a transient decrease of *fli1* expression was observed during 1–2 dpi, but also restored by 3 dpi. *n* = 9. (c) In situ hybridization (ISH) detection of *krt4* expression in the injured fin at 8 hours post injury (hpi) and 2 dpi. There are no detectable changes of *krt4* expression and morphology of the epidermal layer. *n* = 5, respectively. m, fin ray mesenchyme; r, fin ray bone; k, keratinocytes. (d) Detection of apoptosis in the cryoinjured tissue by the TUNEL staining. TUNEL^+^ cells increased in the injured region at 8 hpi, but they were hardly detected at 2 dpi. Tissue section showed that the TUNEL^+^ cells distribute in the fin ray, the basal cells of the epidermis, and the keratinocyte layer. *n* = 5. (e) Quantification of the number of apoptotic cells in the arbitrary 1 mm × 1 mm square area. Apoptotic cells increased 8 hpi, and then decreased by 2 dpi. Data are presented as the mean ± SEM. Statistical significance was analyzed by one‐way ANOVA; ***p < .001. (f) Recruitment of neutrophils, which was visualized by *Tg*(*lysC:egfp*), after cryoinjury. Neutrophils accumulated in the injured region by 8 hpi, but they started to leave from the region at 1 dpi and mostly disappeared at 2 dpi. *n* = 6. Scale bars: 500 μm (a), 100 μm (b, f), 50 μm (c), 100 μm (whole‐mount in d) and 50 μm (section in d). Circles mark the cryoinjured regions.

In this study, we developed a mild cryoinjury method in which local cryoinjury was induced using a fine cooled needle (Figure 3a). After cryoinjury, the damaged area can be identified due to the frost surrounding the cooled area. At 2 dpi, the number of melanocytes in the injured area temporarily decreased, but recovered by 10 dpi (Figure 3a). The decrease in melanocyte number helped to identify the site of damage during the observation period.

In the injured area, hyperemia was often observed at 1 dpi (Figure 3a, arrowhead). To closely observe the effect on blood flow around the cryoinjury site, we used *Tg*(*gata1:mrfp*) (Fukui et al., 2009). At 1 dpi, a severe decrease of blood flow was often observed (Figure 3b, upper left panel; Video S1). However, as early as 2 dpi, the red blood cells began to disperse, and the stagnation of blood cells was dissolved by 3 dpi (Figure 3b, upper right panels; Video S2). When the state of the vascular endothelium was examined using *Tg*(*fli1:egfp*) (Kitaguchi et al., 2009), EGFP expression decreased around the injured area at 1 dpi, but was almost completely recovered by 3 dpi (Figure 3b, lower panels). Hence, cryoinjury caused temporal destruction of the vascular network and blood flow.

In the epidermis, which is composed of a keratinocyte layer and a basal layer in zebrafish (Chen et al., 2011; Liu et al., 2024), no major damage to epidermal layers was observed judging from the *keratin 4* (*krt4*) expression in the superficial keratinocytes and the overall morphology (Figure 3c).

To further examine the damage caused by cryoinjury, cell death was detected by the TUNEL staining. At 8 hours post injury (hpi), a significant increase in apoptosis was detected in the injured area; however, at 2 dpi, only a few apoptotic cells were observed (Figure 3d,e). When we examined the distribution of TUNEL^+^ cells in sections, they were found both in the epidermis and mesenchymal cells within the fin rays. Consistent with cell death, the accumulation of myeloid cells in the cryoinjured region from 8 hpi to 1 dpi was observed by using the *Tg*(*lysC:egfp*) (Kitaguchi et al., 2009) (Figure 3f).

### 3.4 Mild cryoinjury induces RRE response without forming the blastema

**FIGURE 4:**
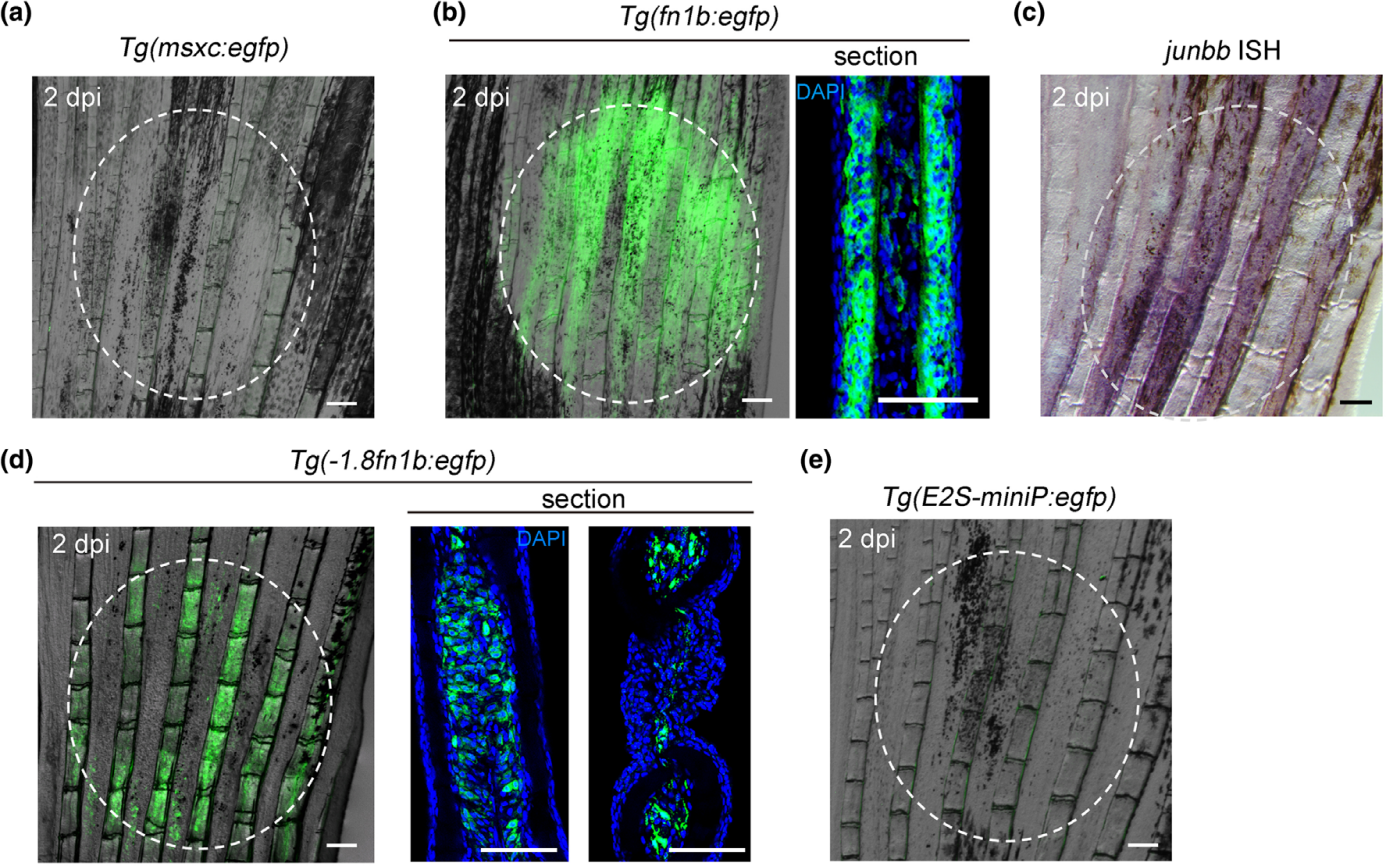
Response of regeneration‐response enhancers (RREs) and regeneration genes after cryoinjury. (a) Absence of *msxc* induction at 2 days post injury (dpi) in the *Tg*(*msxc:egfp*). *n* = 5. (b) Expression of EGFP in the *Tg*(*fn1b:egfp*) at 2 dpi. Tissue section showed that EGFP expression is observed in the epidermal keratinocytes as in the regenerating fin. *n* = 5. (c) Induction of *junbb* at 2 dpi as detected by in situ hybridization (ISH). The ISH signal is seen in the fin ray mesenchymal cells. *n* = 5. (d) Response of *Tg*(*−1.8fn1b:egfp*) to the cryoinjury at 2 dpi. Tissue sections revealed that mesenchymal cells that occupied fin ray expressed EGFP. *n* > 15. (e) Absence of response in the *Tg*(*E2S‐miniP:egfp*) after cryoinjury. *n* = 5. Scale bars, 100 μm (whole‐mount) and 50 μm (section). Dotted circles mark the cryoinjured regions.

We investigated the expression of regenerative genes and RRE responses using the cryoinjury model. Cryoinjury induced mesenchymal *junbb* and epithelial *fn1b* expression, but, *msxc* expression was not induced (Figure 4a–c). The data suggest that the cryoinjury causes the priming of regenerative cells, but it does not induce the blastema formation.

Next, we used RRE Tgs to examine whether cryoinjury induces RRE activation. The activation of *1.8 k* RRE was detected in the injured area (Figure 4d), in which the distribution of EGFP^+^ cells was observed in the mesenchymal cells of the fin rays and inter‐rays. In contrast, the *E2S* RRE was not activated by cryoinjury (Figure 4e), although *fn1b* activation in the epithelium was induced (Figure 4b). We speculated that the absence of *E2S* RRE activation by cryoinjury could be the result of the lower sensitivity of the *E2S* RRE to injury signals.

In summary, we showed that mild cryoinjury is a model that does not cause significant tissue damage or tissue loss. This model is unique as it does not involve blastema formation, but the response of RRE and several regeneration‐induced genes occurs. The cryoinjury model and RRE Tg may provide an excellent platform for exploring the injury signal required for RRE activation. We speculated that cell death and/or accompanying cell reorganization may serve as the injury signal; however, this is a subject for future research.

## AUTHOR CONTRIBUTIONS

Takafumi Yoshida and Atsushi Kawakami designed the experimental strategy, analyzed the data, and prepared the manuscript. All authors have approved the manuscript.

## CONFLICT OF INTEREST STATEMENT

The authors declare no conflicts of interest directly relevant to the content of this article.

## Supporting information


**VIDEO S1:** Blood flow live imaging of *Tg(gata1:mRFP)* at 1 day post cryoinjury (1 dpi). White and yellow arrowheads indicate typical sites of blood congestion and reduced blood flow, respectively.

**VIDEO S2:** Blood flow live imaging of *Tg(gata1:mRFP)* at 3 days post cryoinjury (3 dpi). White and yellow arrowheads indicate the corresponding sites in Video S1, where blood congestion and reduced blood flow were evident at 1 dpi, respectively.

## Data Availability

The data that support the findings of this study are available from the corresponding author upon reasonable request.
